# Sacrum morphometry and spinopelvic parameters among the Indonesian population using computed tomography scans

**DOI:** 10.1097/MD.0000000000027955

**Published:** 2021-11-24

**Authors:** Arsanto Triwidodo, Ahmad Jabir Rahyussalim, Nyimas Diana Yulisa, Jacub Pandelaki, Lina Saleh Huraiby, Ivana Ariella Nita Hadi, Faza Yuspa Liosha, Ismail Hadisoebroto Dilogo

**Affiliations:** a Department of Orthopaedic and Traumatology, Faculty of Medicine Universitas Indonesia, Cipto Mangunkusumo Hospital, Jakarta, Indonesia; b Department of Radiology, Faculty of Medicine Universitas Indonesia, Cipto Mangunkusumo Hospital, Jakarta, Indonesia; c Faculty of Medicine Universitas Indonesia, Jakarta, Indonesia.

**Keywords:** computed tomography scans, Indonesian population, morphometry, sacrum vertebrae, spinopelvic parameters

## Abstract

This is a cross-sectional study. This study aims to describe the characteristics of sacrum vertebrae and spinopelvic parameters among the Indonesian population and compare them with studies from other populations. This study also intends to determine the sexual dimorphism of sacrum vertebrae and find the correlations between spinopelvic parameters.

Morphometry of the sacrum is necessary for designing sacral prosthesis and instrumentations. Knowledge of spinopelvic parameters further supports the prosthesis installation procedure to restore the physiological spinal alignment of the patients. However, previous studies showed varied results among different populations. This is the first study to be conducted among the Indonesian population.

Morphometric dimensions of sacrum vertebrae and the spinopelvic parameters (pelvic incidence, pelvic tilt, sacral slope, lumbar lordosis) were analyzed using thin-cut (1 mm) computed tomography images in 150 males and 150 females, aged 25 to 50 years without any spinal pathology.

Generally, the size of the sacrum vertebrae was greater in males (*P* < .05). The sacral index, curvature index, and corporo-basal index were statistically different between genders (*P* < .001). Lumbar lordosis was the only spinopelvic parameter found significantly greater in females (*P* < .001). Significant positive correlations between all spinopelvic parameters, except for lumbar lordosis and pelvic tilt, were found in the present study (*P* < .001).

The study serves as the first large series database of sacrum morphometric characteristics and spinopelvic parameters of the Indonesian population. There was significant gender-associated differences in various dimensions of sacrum vertebrae. The sacral index was found to be the most useful parameter for sex determination. There were strong significant positive correlations between various spinopelvic parameters. A comparison of populations revealed morphometric characteristic differences, which is proved to be critical in surgical implications.

## 1 Introduction

Sacrum morphometry and spinopelvic parameters are vital for various spine surgical interventions and understanding biomechanical aspects.^[1]^ The sacrum is the only skeletal connection between the trunk and the lower body provides stability and strength for the pelvis, as it is wedged between the 2 hips. This allows the sacrum to endure and transmit weight equally to the pelvis and the lower extremities.

In clinical settings, spinopelvic reconstruction post-sacrectomy, both total and partial, is widely used in the treatment of malignancies, traumas, infections (sacral tuberculosis), and degenerative diseases.^[2–4]^ Spinopelvic reconstruction using prosthesis and implants may overcome spinopelvic instability to alleviate pain, promote early ambulation, and fill in the dead cavity (caused inevitably by the wide resection), and thus preventing sacrococcygeal herniation and the risk of infections and wound dehiscence.^[5,6]^ However, the anatomical complexity of the bone remains a challenge in developing accurate prosthesis and installation technique, thus, requires detailed knowledge of the sacrum anatomy. Moreover, the emphasis on spinopelvic parameters is essential to show the normal orientation of the spinopelvic region to restore the physiological spinal alignment of the patients.^[7,8]^

Further, other potential use of sacrum morphometry includes sex determination in forensic through sexual dimorphism, which is necessary to make age, ancestry, and stature estimations.^[9,10]^ Besides, spinopelvic parameters can be a useful guide to evaluate instability in patients with chronic low back pain for planning operative management.^[11]^

Several studies demonstrated that sacrum morphometry varied among ethnicities.^[1,12]^ This was the first study among the Indonesian population to describe the morphological characteristics of sacrum vertebrae and spinopelvic parameters using computed tomography (CT)-Scan images, reporting at a national referral hospital where patients from all across the country are referred. The present study also aims to compare the differences between males and females and other populations. Further, our study intends to show correlations between spinopelvic parameters.

## 2 Materials and methods

This was a cross-sectional study on CT Scan images of the sacrum vertebrae and the spinopelvic parameters of 150 males and 150 females. The study was conducted in Cipto Manungkusumo National Referral Hospital, which is presumed to represent all the ethnicities of the Indonesian population. The samples were collected retrospectively from January to July 2020. The age range of the patients was 25 to 50 years, where a complete fusion of vertebral epiphyseal plate has been achieved; meanwhile, the degenerative process has not begun. The exclusion criteria of our samples were vertebral pathologies such as fractures, congenital anomalies (e.g., hemivertebrae), tumor, and other pathologies. This study has been approved by the Ethics Committee of Faculty of Medicine, Universitas Indonesia with protocol number registered 20-05-0528. Informed consents were not retrieved in this study as it is not mandatory for our study according to the Ethics Committee because all the CT images were collected retrospectively from the hospital database. Besides, all the morphometric results presented were accumulation measurements of all the images, thus all the data remain anonymous.

The digital imaging and communications in medicine (DICOM) Viewer, PACS 3.0.11.5 (INFINITT Healthcare Co, Ltd, South Korea), was used to measure the thin-cut (1 mm) Abdominal CT Scan images (using CT Scan SIEMENS SOMATOM DEFINITION FLASH dual source 128-slice with 120 kV, auto-mAs or CT Scan PHILIPS INGENUITY 64-slice with 100 kV, auto-mAs). An interobserver error was not assessed in this study as measurements were done by a single investigator. Measurements were recorded in millimeters (mm) and degree (^o^).

**Figure 1:**
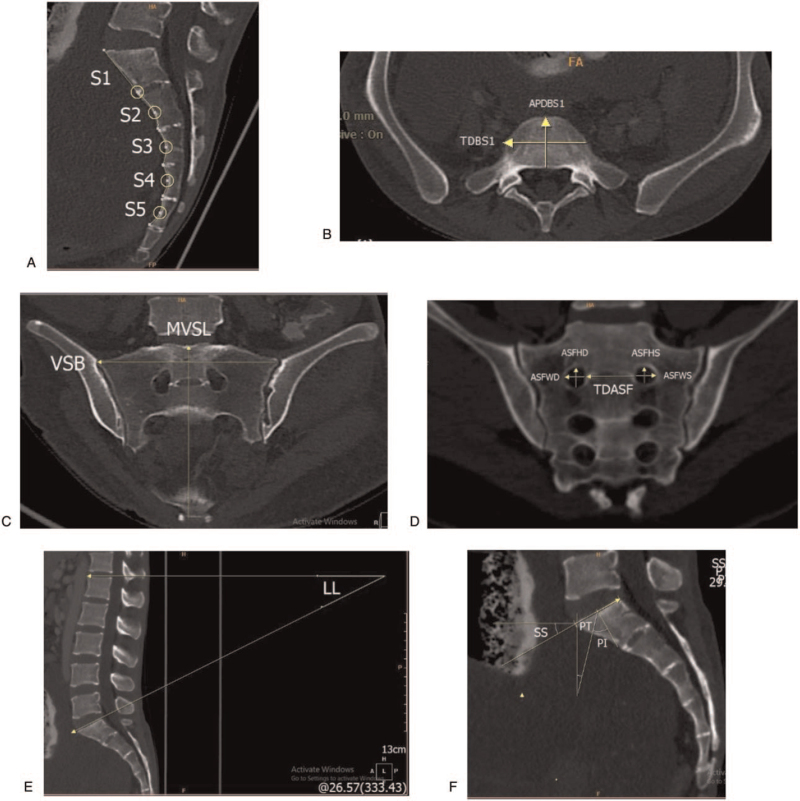
(A) The sacral bodies (S1-S5) curved length. (B) The anterior-posterior and the transverse diameter of the first sacral body. (C) The mid-ventral straight length and the ventral straight breadth. (D) The anterior sacral foramina of the first sacral body right and left; the transverse distance between the sacral foramina. (E) The lumbar lordosis (LL). (F) The sacral slope (SS); the pelvic tilt (PT); the pelvic incidence (PI).

There were various anatomical parameters of the sacrum measured in this study adopted from previous studies as described below.^[1,10]^ From the lateral images, we measured the length of the S1 vertebral body defined as the curved length between the midline of the promontory and the transverse fusion line of the sacral foramina measured in the sagittal plane (Fig. 1A). We also measured the curved length of each of the S2,S3,S4,S5 vertebral body, which altogether made the total curved length S1-S5 (Fig. 1A).

Two measurements of the first sacral body were made on the axial plane (Fig. 1B). The distance along the midline between the sacral promontory and the posterior border of the first sacral vertebral body was measured to obtain the anterior-posterior diameter of the body of the first sacral vertebra (APDBS1). In addition, the width between the most lateral points of the first sacral vertebral body known as the transverse diameter of the body of the first sacral vertebra (TDBS1).

On the coronal-section, we measured 5 dimensions. The parameter measured included the straight length from the sacral promontory along the midline to the anterior margin of the last sacral vertebra, termed the mid-ventral straight length (MVSL) (Fig. 1C). We also measured the ventral straight breadth (VSB), which was the maximum linear ventral distance between the margins of the lateral wings (Fig. 1C). The first anterior sacral foramina height sinistra/dextra (ASFHS / ASFHD) was measured as the maximum vertical distance between the superior and the inferior borders of the first anterior sacral foramina, whereas the first anterior sacral foramina width dextra/sinistra (ASFWS/ASFWD) was defined as the maximum transverse distance between the medial and lateral borders of the first anterior sacral foramina. The transverse distance between the medial borders of the first anterior sacral foramina at the level of the transverse fusion line of the first anterior sacral foramina was termed as the transverse distance between the first anterior sacral foramina (TDASF) (Fig. 1D). According to the measurements above, 4 indices were calculated using the following formula as summarized in Table 1.

**Table 1:** The equations of the indices of the sacral vertebrae.

Parameter	Formula
Sacral Index	Ventral Straight Breadth × 100÷Mid-Ventral Straight Length
Curvature Index	Mid-Ventral Straight Length ×100 ÷S1-S5 Curved Length
Corporo-Basal Index	Transverse Diameter of the Body S1 ×100 ÷Transverse Diameter of the Base
Index of body of first sacral vertebrae	Anterior-Posterior Diameter of Body S1 ×100 ÷Transverse Diameter of the Body S1

The spinopelvic parameters were measured on the sagittal plane as shown below. The first parameter is the lumbar lordosis (LL), the angle from the superior endplate of the first lumbar to the superior endplate of the first sacral vertebra (Fig. 1E). The sacral slope (SS) was defined as the angle between the superior endplate of the first sacral vertebra and the line from the horizontal axis. The angle between the line perpendicular to the superior endplate of the first sacral vertebra at its midpoint and the line connecting this point to the center of the femoral head (bicoxofemoral axis) made the pelvic incidence (PI). Lastly, the measurement of the pelvic tilt (PT) was defined as the angle between the line connecting the midpoint of the first sacral vertebral endplate to the axis from the femoral head and the vertical axis. The SS, PI, and PT are shown in Fig. 1F.

The data were analyzed using IBM SPSS Statistics 20 software with a *P*-value of ≤.05 to be deemed significant. All data collected were analyzed descriptively to obtain the mean, standard deviation, and median (minimum – maximum) and to obtain statistical differences between males and females using independent *t* test or Mann–Whitney *U* test. Moreover, correlations between spinopelvic parameters were analyzed using Pearson or Spearman.

## 3 Results

Table 2 summarizes the results of the sacrum dimensions of 150 males and 150 females with a median of age 35 (25–50) years. The sacral body decreased gradually from the first body to the fourth body and remained constant afterward in the last sacral body. The total curved length had a mean of 112.53 mm ± 7.98 mm, while the mid-ventral straight length was not normally distributed with the median of 104.7 mm (75.1 129.12 mm). Both the curved and straight length were greater in males (*P* = 01; *P* < .001, respectively). The ventral straight breadth, however, was found to be greater in females (*P* *<* .001). We found that in the first sacral body, the transverse diameter was greater than the anterior-posterior diameter.

**Table 2:** Gender-associated differences of the sacrum dimensions and indices.

	The Sacrum Dimensions and Indices			
Parameter	Male + Female	Male (n = 150)	Female (n = 150)	*P*
Sacral 1 Height	30.59 ± 2.39	30.80 ± 2.39	30.37 ± 2.38	.12^†^
Sacral 2 Height	25.66 ± 2.57	26.15 ± 2.59	25.16 ± 2.45	<.001^∗,†^
Sacral 3 Height	20.31 ± 2.49	20.52 ± 2.55	20.10 ± 2.42	.14^†^
Sacral 4 Height	17.94 ± 2.05	18.07 ± 2.01	17.80 ± 2.08	.27^†^
Sacral 5 Height	17.96 ± 2.37	17.99 ± 2.51	17.92 ± 2.22	.79^†^
Sacral 1–5 Curved length	112.53 ± 7.98	113.69 ± 7.76	111.36 ± 8.05	.01^∗,†^
Mid-ventral straight length	104.7 (75.1–129.12)	107.12 (86.62–129.12)	104.31 (75.1–121.99)	<.001^∗,‡^
Ventral straight breadth	107.16 ± 5.90	105.70 ± 5.72	108.62 ± 5.73	<.001^∗,†^
Transverse diameter of the body sacral 1	49.98 ± 4.04	52.06 ± 3.41	47.90 ± 3.53	<.001^∗,†^
Anterior-posterior diameter of body sacral 1	32.43 ± 2.93	34.01 ± 2.69	30.85 ± 2.22	<.001^∗,†^
Anterior sacral foramina height dextra	14.37 ± 2.12	14.17 ± 1.89	14.56 ± 2.31	.12^†^
Anterior sacral foramina width dextra	11.22 ± 1.59	10.91 ± 1.49	11.52 ± 1.64	<.001^∗,†^
Anterior sacral foramina height sinistra	14.47 ± 1.99	14.29 ± 1.85	14.65 ± 2.11	.12^†^
Anterior sacral foramina width sinistra	11.23 ± 1.49	11.05 ± 1.51	11.41 ± 1.46	.04^∗,†^
Transverse distance between anterior sacral foramina 1	28.28 (21.28–40 72)	29.25 (22.06–40.72)	27.42 (21.28–39.56)	<.001^∗,‡^
Sacral index	102.44 (75.46–134.11)	98.89 (75.46–129.45)	104.18 (88.2–134.11)	<.001^∗,‡^
Curvature index	94.69 (81.48–119.8)	93.82 ± 3.16	97.30 (84.26–119.8)	<.001^∗,‡^
Corporo-basal index	47.10 ± 4.47	49.58 ± 3.58	44.61 ± 3.84	<.001^∗,†^
Index of body sacral 1	65.02 ± 4.95	65.43 ± 4.79	64.62 ± 5.10	.15^†^

Values are presented in mean ± standard deviation, or median (minimum – maximum), *P* ≤ .05^∗^ is statistically significant. Data were recorded in millimeters (mm). Data were analyzed using Independent *t* test^†^, Mann--Whitney^‡^.

The right and left anterior sacral foramina of the first sacral body had similar height and width. However, the height was greater than the width in both ASCF. The distance between the 2 ASCF was not distributed evenly with the median of 28.28 mm (21.28–40.72 mm). In all of the sacrum vertebrae dimensions, males were greater than females except for the VSB and ASCFW. The sacral index and the curvature index were significantly greater in females, whereas the corpora-basal index was greater in males (*P* *<* .001) (Table 2).

The spinopelvic parameters are demonstrated in Table 3. The LL had a mean of 38.72^o^ ± 9.85^o^ and significantly greater in females (*P* *<* .001). In all of the other parameters, that is, the SS, the PI, and the PT, the values were greater in females, but none of them showed significant difference (*P* = 06; .05; .19, respectively). There was significant strong positive correlation between LL/PI and PI/SS (*r* = *.63* and *r* = *.73,* respectively, with *P* < .001). Moreover, a very strong positive correlation was found between LL and SS (*r* = 0.87*, P* *<* .001*).* Besides, PI/PT showed moderate positive correlation (*r* = 0.60, *P* < .001) (Table 4).

**Table 3:** Gender-associated differences of the spinopelvic parameters.

	The Spinopelvic Parameters			
Parameter	Male + Female	Male (n = 150)	Female (n = 150)	*P*
Sacral slope	34.96 ± 7.08	34.17 ± 6.69	35.74 ± 7.39	.06^†^
Pelvic incidence	50.16 ± 9.18	49.13 ± 8.90	51.20 ± 9.37	.05^†^
Pelvic tilt	14.7 (1.15–34.16)	13.93 (1.55–33)	15.27 (1.15–34.16)	.19^‡^
Lumbar lordosis	38.72 ± 9.85	35.76 ± 8.92	41.68 ± 9.88	<.001^∗,†^

Values are presented mean ± standard deviation, or median (minimum – maximum), *P* ≤ .05^∗^ is statistically significant. Data were recorded in degrees (^o^). Data were analyzed using independent *t* test^†^, Mann--Whitney^‡^.

**Table 4:** Correlations between spinopelvic parameters.

	Correlation Coefficient (*r*-value)		
Parameter	Male + Female	Male (n = 150)	Female (n = 150)
LL/PI^†^	0.631^∗^	0.675^∗^	0.563^∗^
LL/SS^†^	0.869^∗^	0.897^∗^	0.810^∗^
LL/PT^‡^	−0.046	−0.020	−0.125
PI/PT^‡^	0.596^∗^	0.596^∗^	0.585^∗^
PI/SS^†^	0.729^∗^	0.757^∗^	0.695^∗^

∗ *P* < .001. Data were analyzed using Pearson^†^ and Spearman^‡^. Lumbar lordotic (LL); Pelvic incidence (PI); Sacral slope (SS); Pelvic tilt (PT).

## 4 Discussion

In the present study, we provide comprehensive morphometric characteristics of the sacrum and spinopelvic parameters of the Indonesian population with special regard in designing sacral prosthesis and installation technique. The results showed the sexual dimorphism of several dimensions. We also found morphological differences between the Indonesian population and other populations. Further, there were correlations between spinopelvic parameters.

The total curved length was higher in males than in females (*P* = .01). These results were aligned with the Oman population, which, however, reported higher values in the total curved length of the males.^[10]^ However, the present study showed higher values of mid-ventral straight length compared with other studies, especially in females, as summarized in Table 5.^[9,10,12,13]^ We found comparable results of the first sacral body height (mean value of 30.59 mm ± 2.39 mm) with the Indian, Turkish, and Mexican populations.^[1,10,14,15]^ According to Tomita et al, sacrectomy can be classified based on the level of sacral amputation.^[16]^ Thus, the sacral bodies curved length (S1-S5) and the total curved length would provide information in designing prosthesis for partial reconstruction based on the sacral level excised.

**Table 5:** A comparison of populations of the sacral vertebrae dimensions.

										ASFH		ASFW		
References	Population	Gender	N	MVSL	S1-S5 curved length	VSB	Sacral 1 body height / length	APDBS1	TDBS1	Right	Left	Right	Left	TDSAF
Kumar et al and Vishwakarma^[10]^	Oman	Male	21	102.7 ± 4.9	116.4 ± 4.0	99.9 ± 7.3	–	30.6 ± 1.4	53.0 ± 3.3					
		Female	22	93.5 ± 6.4	110.7 ± 6.7	109.5. ± 3.7	–	30.5 ± 0.9	51.4 ± 2.2					
Mazumdar et al^[13]^	Indian	Male	127	100.8 ± 11.5	108.2 ± 6.7	–	–	29.4 ± 3.8	41.6 ± 8.5					
		Female	123	87.3 ± 7.4	99.3 ± 7.4	–	–	27.9 ± 2.7	39.7 ± 5.2					
Shingare et al^[9]^	Indian	Male	25	104.73 ± 12.6	–	102.93 ± 4.83	–	–	–					
		Female	25	92.64 ± 6.1	–	104.77 ± 6.48	–	–	–					
Basaloglu et al^[12]^	Turkish	Male	30	103.1 ± 1.13	–	102.2 ± 7.0	30.6 ± 3.1	31.7 ± 3	52.7 ± 6.1					
		Female	30	102.0 ± 1.02	–	108.4 ± 6.0	29.8 ± 2.4	30.3 ± 2.8	52.6 ± 7.9					
Hussein et al^[17]^	Egyptian	Male	109	–	–	108.3 ± 7.6.0	–	42.4 ± 5.23	54.79 ± 6.0					
		Female	91	–	–	107.59 ± 6.3	–	40.57 ± 5.5	51.71 ± 5.1					
Avalos Morales et al^[15]^	Mexican	Male + Female	50	–	–	–	31.11 ± 2.80	31.93 ± 2.91	48.72 ± 4.64	13.72 ± 2.03	13.6 ± 2.11	13.02 ± 3.11	13.28 ± 2.67	32.33 ± 41.9
Saluja et al^[1]^	Indian	Male + Female	108	–	–	–	29.62 ± 2.65	30.04 ± 2.50	47.64 ± 4.98	12.95 ± 1.65	12.99 ± 1.59	12.28 ± 1.69	12.18 ± 1.8	30.10 ± 3.19
Arman et al^[14]^	Turkish	Male + Female	100	–	–	–	30.22 ± 2.35	31.42 ± 2.83	49.40 ± 5.89	13.58 ± 2.16	13.74 ± 2.0	13.78 ± 2.12	14.13 ± 2.10	30.48 ± 2.78
Franklin et al^[29]^	Australian	Male+ Female	400	108.1 ± 10.72	–	101.9 ± 7.34	–	–	46.9 ± 5.92					
Present Study	Indonesian	Male	150	107.12 (86.62–129.12)	113.69 ± 7.76	105.70 ± 5.72	30.80 ± 2.39	34.01 ± 2.69	52.06 ± 3.41	14.17 ± 1.89	14.29 ± 1.85	10.91 ± 1.49	11.05 ± 1.51	29.25 (22.06–40.72
		Female	150	104.31 (75.1–121.99)	111.36 ± 8.05	108.62 ± 5.73	30.37 ± 2.38	30.85 ± 2.22	47.90 ± 3.53	14.56 ± 2.31	14.65 ± 2.11	11.52 ± 1.64	11.41 ± 1.46	27.42 (21.28–39.56)

Data were recorded in millimeters (mm). Values are presented as mean ± SD or median (minimum - maximum) if data were not distributed evenly. Mid-ventral straight length (MVSL); Sacral 1–5 curved length (S1-S5 curved length); Ventral straight breadth (VSB); Anterior-posterior diameter of body sacral 1 (APBDBS1); Transverse diameter of body sacral 1 (TDBS1); Anterior sacral foramina height (ASFH); Anterior sacral foramine width (ASFW); Transverse distance between anterior sacral foramina (TDASF).

The first level of the sacrum forms an interface with the pelvic known as the sacroiliac joint. The sacroiliac joint connects the spine and the pelvic serves as a bridge of the upper body and the lower body. A prosthesis should be embedded suitably to establish an adequate interface to transmit bodyweight evenly. Thus, we evaluated the maximum linear distance between the margins of the 2 lateral wings (the ventral straight breadth). Our data reported that the maximum ventral breadth of the sacrum was wider in females (*P* *<* .001) and the values varied slightly among studies (summarized in Table 5); however in the Egyptian population, no significant differences were found between males and females (*P* = .504).^[9,10,12,17]^

The APDBS1 showed lesser results than the TDBS1. Both were found to be significantly greater in males (*P* *<* .001). These results were consistent with the study in Oman populations that found the first sacral body to be sexually dimorphic, although unlike in the present study, they did not show the statistically significant result.^[10]^ The Egyptian reported the highest results (with mean difference ± 10 mm) compared with other populations as summarized in Table 5.^[17]^ The dimensions of the anterior-posterior diameter and the transverse diameter of the first sacral body are essential to establish an accurate lumbosacral interface during the anterior and posterior lumbar body fusion.

The sacral foramina serve as the exit of the anterior divisions of the sacral nerves and act as the entrance of the lateral sacral arteries. The nerve preservation during sacrectomy remains a challenge due to the anatomical complexity, however, significantly contributes to the improvements in quality of life of the patients. The procedure allows the preservation of essential functions of bladder and bowel control, sexual function, and other motoric and sensory functions.^[18]^ Thus, the design of the prosthesis in the reconstruction should meet the demands required for nerve preservation, which is further facilitated by the morphometry characteristics of the sacral foramina. In addition, these dimensions are essential during sacral nerve roots electric stimulation for lower urinary tract neural reflex modulation.^[1]^ The first anterior sacral foramina of the Indonesian population were slightly oval, with the mean value of the height of 14.37 mm (right) and 14.47 (left); and the mean of the width of 11.22 mm (right) and 11.23 mm (left). On the contrary, the sacral foramina were found in a round shape in several other studies, which however showed similar results with our study (summarized in Table 5). The distance between the 2 first anterior sacral foramina had a mean of 28.28 mm (21.28 – 40.72), which is slightly lower than the Turkish, Indian, and Mexican populations.^[1,14,15]^

There were 4 indices measured in this study calculated using the formulae described above. These indices were important to show sexual dimorphism. All the indices were statistically significant between both genders (*P* *<* .001), except for the index of the first sacral body (*P* = .154). The sacral index and the curvature index were found to be greater in females, whereas the others were greater in males. Our results supported several previous studies suggesting the sacral index as the most significant parameter for sex determination of the bone (Table 6).^[9,10,13]^ Further, Shingare et al reported that 57.9% of bones in females and 27.71% of bones in males were identified using this parameter.^[9]^

**Table 6:** Comparison of populations of the indices of the sacrum.

References	Population	Gender	N	Sacral Index	*P*	Curvature Index	*P*	Corporo-basal Index	*P*	Index of the body sacral vertebrae 1	*P*
Kumar and Vishwakarma^[10]^	Oman	Male	21	97.51 ± 8.15	<.001^∗^	88.31 ± 3.63	<.001^∗^	49.6 ± 1.44	<.001^∗^	57.94 ± 4.38	.15
		Female	22	117.35 ± 5.72		84.44 ± 2.72		43.93 ± 2.26		59.63 ± 3.11	
Mazumdar et al^[13]^	Indian	Male	127	94.9 ± 4.8	<.001^∗^	94 ± 2.7	<.001^∗^	43.8 ± 9.1	.02^∗^	71.6 ± 9.1	.35
		Female	123	109.8 ± 7.3		87.9 ± 4.2		41.7 ± 3.3		70.7 ± 5.8	
Shingare et al^[9]^	Indian	Male	30	98.44 ± 4.69	<.001^∗^	–	–	–	–	–	–
		Female	30	113.23 ± 5.61		–		–		–	
Present Study	Indonesian	Male	150	98.89 (75.46–129.45)	<.001^∗^	93.82 ± 3.16	<.001^∗^	49.58 ± 3.58	<.001^∗^	65.43 ± 4.79	.15
		Female	150	104.18 (88.2–134.11)		97.30 (84.26–119.8)		44.61 ± 3.84		64.62 ± 5.10	

Data were recorded in millimeters (mm), *P* ≤ .05^∗^ is statistically significant. Values are presented as mean ± SD or median (minimum – maximum), if data were not distributed evenly.

The spinopelvic dimensions are useful to elaborate on the shape and orientation of each closely related anatomical segment in order to maintain a stable posture with minimal energy expenditure. This is important to achieve appropriate spinal alignment during the prosthesis installation procedure.^[7]^ It is commonly evaluated in erect position using the X-ray. In the present study, however, the measurements were conducted using a CT Scan for morphometric study purposes. A study by Chevilotte et al who compared pelvic parameters in standing and supine position reported that PI did not show changes in both positions; LL and PT were smaller, whereas the SS was higher at supine. However, the results did not show clinically significant differences in both positions.^[19]^ In the present study, females showed a greater lordotic angle than males (*P* *<* .001). Other spinopelvic parameters did not show significant differences between genders. The results of the present study were similar to previous studies with a mean difference less or equal to 5 degrees for PI, PT, and SS (Table 7).^[11,20–25]^ However, the LL was found to have the largest mean difference with other studies, although our result (mean value of 38.72^o^ ± 9.85^o^) was closest to that of the Chinese population (mean value of 36.7^o^ ± 11.8^o^).^[21]^ We found significant strong positive correlations between LL/PI, PI/SS, and a very strong correlation between LL and SS. PI and PT also showed significant moderate positive correlation (Table 4). In other words, a directly proportional relationship was observed between the mentioned parameters. These results were similar with other studies and Chevilotte et al, which further stated that LL/PI, LL/SS, and PI/PT showed strongest correlations in supine position that might be due to slight hyperextension of the hip and consequently may increase SS and limit the extension of lumbar spine.^[19–21]^ However, Mac-Thiong et al showed significant weak negative correlation between PI/SS (*r* = −.19, *P* *<* .001).^[25]^

**Table 7:** Comparison of populations of the spinopelvic parameters.

References	Population	N	Pelvic incidence	Pelvic tilt	Sacral slope	Lumbar lordosis
Lee et al^[24]^	Korean	86	47.8 ± 9.3	11.5 ± 5.3	36.3 ± 7.8	36.8 ± 7.6
Mac-Thiong et al^[25]^	Canadian	737	52.6 ± 10.4	13.0 ± 6.8	39.6 ± 7.9	–
De Rezende Pratali et al^[23]^	Brazilian	50	48.7 ± 9.6	12.15 ± 6.2	38 ± 8.4	–
Muthuuri et al^[11]^	Kenya	68	55.2 ± 11.9	21.6 ± 15.3	41.3 ± 12.3	41.9 ± 22.4
Hasegawa et al^[22]^	Japanese	126	52.3 ± 11.1	11.5 ± 7.6	40.8 ± 8.5	40.4
Zeng et al^[21]^	Chinese	85	51.1 ± 8.2	18.5 ± 8.0	32.8 ± 6.3	36.7 ± 11.8
Chevillote et al^[19]^	American	15	49.3 ± 8.1	12.1 ± 6.3	37.1 ± 6.3	54.8 ± 9.8
Bhosale et al^[20]^	India	130	51.5 ± 6.85	12.32 ± 5.41	39.17 ± 6.26	–
Present Study	Indonesian	300	50.16 ± 9.18	14.7 (1.15–33)	34.96 ± 7.08	38.72 ± 9.85

Data were recorded in degree (^o^). Values are presented as mean ± SD or median (minimum – maximum), if data were not distributed evenly.

Failure to restore spinal alignment during lumbar fusion may cause pain postoperatively and accelerate adjacent segment degeneration.^[26]^ Besides, reduced LL may cause diminishing of tension of the anterior soft tissue structures and consequently increase the load on the posterior spinal implant and its interface with the vertebrae, thus associated with poor clinical outcomes.^[27]^ Spinopelvic parameters abnormality, especially PI, also contribute to the pathological process of isthmic spondylolysis, spondylolisthesis, various degenerative diseases (including lumbar degenerative disease, low back pain, hip osteoarthritis), spinal deformity, fixed sagittal imbalance (FSI), and adolescent idiopathic scoliosis (AIS). PI is regulated by an acceptable range of values of LL to maintain sagittal balance. Moreover, increase PT values reflect pelvic retroversion can also be a marker of the spinopelvic alignment imbalance.^[28]^ Thus, in surgical interventions of the spine, an adequate LL should be maintained for the given spinopelvic parameters. As an example, patients with elevated PI would demand elevation in LL and vice versa.^[20]^ Further, a balanced spinopelvic alignment should demonstrate direct relationship between PI, SS, and LL as also shown in our study.^[28]^

The study has strengths and limitations. This study serves as the first reliable database of sacrum morphometry and spinopelvic parameters with large data series of 300 sacrum vertebrae. Morphometric measurements using CT Scan images have both advantages and disadvantages. For reconstruction post-sacrectomy purposes, a good acquaintance of CT Scan anatomy preoperatively allows identifying the morphology of the sacrum vertebrae and spinopelvic parameters, which would assist the prosthesis designing process and the installation procedure. Thus, virtual studies using CT Scan would represent the clinical setting. Further, the age groups of our study were determined to obtain the normal bones without any potential degenerative process. Several other studies also supported the method for morphometric measurements using CT Scan.^[17,29,30]^ However, the other aforementioned studies used dry bones from the anatomy banks.^[1,9,10,14]^ We are aware that dry bones allow a 3-dimensional view of the bones, unlike CT Scan. However, a comparison study between the 2 measurement tools, the CT and the caliper for sacrum morphometry by Zech et al in 2012 on 95 patients of the Swiss population showed no significant differences between both groups, thus supported the use of CT Scan.^[30]^ Besides, obtaining the samples of dry bones of a certain determined age groups would be posed another challenge. Thus, the method using CT Scan images for morphometry study is considerable in the forthcoming studies.

## 5 Conclusion

This study found significant differences in various dimensions of sacrum vertebrae and the spinopelvic parameters between males and females. The sacral index was found to be the most significant parameter for sex determination. LL was highly varied among populations. Significant positive correlations between various spinopelvic parameters were also found. Moreover, a comparison of populations showed differences in several sacrum dimensions and spinopelvic parameters. These differences have critical implications for surgical purposes.

## Author contributions

A.T., N.D.Y., J.P., and A.J.R. conceived the study; A.T. acquired the data; I.A.N.H, and

L.S.H. conducted the data analyses and interpretation; A.T. and I.A.N.H. wrote the manuscript, A.J.R., I.H.D., N.D.Y., J.P., F.Y.L. participated in discussions and revising the paper critically, funding acquisition A.J.R, A.T., and I.A.N.H.; F.Y.L. and I.A.N.H. responsible for the administrative requirements for ethical approval admission and data acquisition permission; I.H.D., A.J.R., J.P., and N.D.Y. supervised the study. All authors have read and agreed to the published version of the manuscript.

**Conceptualization:** Arsanto Triwidodo, Ahmad Jabir Rahyussalim, Nyimas Diana Yulisa, Jacub Pandelaki.

**Data curation:** Arsanto Triwidodo, Lina Saleh Huraiby, Ivana Ariella Nita Hadi.

**Formal analysis:** Lina Saleh Huraiby, Ivana Ariella Nita Hadi.

**Funding acquisition:** Arsanto Triwidodo, Ahmad Jabir Rahyussalim, Ivana Ariella Nita Hadi.

**Investigation:** Arsanto Triwidodo.

**Methodology:** Ahmad Jabir Rahyussalim, Nyimas Diana Yulisa, Jacub Pandelaki, Ivana Ariella Nita Hadi.

**Project administration:** Ivana Ariella Nita Hadi, Faza Yuspa Liosha.

**Resources:** Jacub Pandelaki.

**Supervision:** Ahmad Jabir Rahyussalim, Nyimas Diana Yulisa, Jacub Pandelaki, Ismail Hadisoebroto Dilogo.

**Writing – original draft:** Arsanto Triwidodo, Ivana Ariella Nita Hadi.

**Writing – review & editing:** Ahmad Jabir Rahyussalim, Ismail Hadisoebroto Dilogo, Faza Yuspa Liosha, Jacub Pandelaki, Nyimas Diana Yulisa.

## References

[1] Saluja S Agarwal S Tuli A. Morphometric analysis of the sacrum and its surgical implications. J Clin Diagnostic Res 2018;7:6064–70.

[2] Li Z Lv Z Yang Z. One-step reconstruction with a novel suspended, modular, and 3D-printed total sacral implant resection of sacral giant cell tumor with preservation of bilateral S1–3 nerve roots via a posterior-only approach. Orthop Surg 2019;12:58–66. 31854115 10.1111/os.12582 PMC7031587

[3] Wei R Guo W Ji T. One-step reconstruction with a 3D-printed, custom-made prosthesis after total en bloc sacrectomy: a technical note. Eur Spine J 2017;26:1902–9. 27844229 10.1007/s00586-016-4871-z

[4] Kim D Lim JY Shim KW. Sacral reconstruction with a 3D-printed implant after hemisacrectomy in a patient with sacral osteosarcoma: 1-year follow-up result. Yonsei Med J 2017;58:453–7. 28120579 10.3349/ymj.2017.58.2.453 PMC5290028

[5] Kiatisevi P Piyaskulkaew C Kunakornsawat S. What are the functional outcomes after total sacrectomy without spinopelvic reconstruction? Clin Orthop Relat Res 2017;475:643–55. 26911974 10.1007/s11999-016-4729-z PMC5289156

[6] Maricevich M Maricevich R Chim H. Reconstruction following partial and total sacrectomy defects: an analysis of outcomes and complications. J Plast Reconstr Aesthetic Surg 2014;67:1257–66. 10.1016/j.bjps.2014.05.001 24907194

[7] Tirta YP Saleh I. Correlation between sagittal spinopelvic parameters and Oswestry Disability Index after thoracal and lumbar spine stabilization and fusion. eJournal Kedokt Indones 2017;5:34–7.

[8] Ames CP Smith JS Scheer JK. Impact of spinopelvic alignment on decision making in deformity surgery in adults: a review. J Neurosurg Spine 2012;16:547–64. 22443546 10.3171/2012.2.SPINE11320

[9] Shingare AK Masaram NB Dhapate SS. Morphometric analysis of human sacra. MedPulse Int J Anat 2017;3:34–7.

[10] Kumar A Vishwakarma N. An anthropometric analysis of dry human sacrum: gender discrimination. Int J Sci Res 2015.

[11] Muthuuri JM Surgeon CO. Assessment of spino-pelvic morphometry, a predictor of lumbosacral instability 2016;10:45–50.

[12] Başaloğlu H Turgut M Taşer FA. Morphometry of the sacrum for clinical use. Surg Radiol Anat 2005;27:467–71. 16211321 10.1007/s00276-005-0036-1

[13] Mazumdar S Ray A Mazumdar A. Sexual dimorphism and regional difference in size of sacrum: a study in Eastern India. Al Ameen J Med Sci 2012;5:298–307.

[14] Arman C Naderi S Kiray A. The human sacrum and safe approaches for screw placement. J Clin Neurosci 2009;16:1046–9. 19442524 10.1016/j.jocn.2008.07.081

[15] Morales-Ávalos R Leyva-Villegas JI Vílchez-Cavazos F. Morphometric characteristics of the sacrum in Mexican population. Its importance in lumbosacral fusion and fixation procedures. Cir Cir 2012;80:528–35. 23336147

[16] Tomita K Hata M Tsuchiya H. Watkins RG. En Bloc Sacrectomy BT: Surgical Approaches to the Spine. New York, NY: Springer New York; 2003. 205–16.

[17] Hussein RF Shokry DA Ismail MM. Sex identification from radiologic anthropometry of sacral and fifth lumbar vertebral measurements. Egypt J Forensic Sci Appl Toxicol 2016.

[18] van Wulfften Palthe ODR Houdek MT Rose PS. How does the level of nerve root resection in en bloc sacrectomy influence patient-reported outcomes? Clin Orthop Relat Res 2017;475:607–16. 26992721 10.1007/s11999-016-4794-3 PMC5289168

[19] Chevillotte T Coudert P Cawley D. Influence of posture on relationships between pelvic parameters and lumbar lordosis: comparison of the standing, seated and supine positions. A preliminary study. Rev Chir Orthop Traumatol 2018;104:399–402. 10.1016/j.otsr.2018.06.005 30009961

[20] Bhosale S Pinto D Srivastava S. Measurement of spinopelvic parameters in healthy adults of Indian origin – a cross sectional study. J Clin Orthop Trauma 2019. 03–8. 10.1016/j.jcot.2019.07.013 PMC7452199 32879575

[21] Zeng Z Hai Y Bi Y. Characteristics of sagittal spinopelvic alignment in asymptomatic Han Chinese adults. Exp Ther Med 2018;16:4107–33. 30344686 10.3892/etm.2018.6680 PMC6176135

[22] Hasegawa K Okamoto M Hatsushikano S. Normative values of spino-pelvic sagittal alignment, balance, age, and health-related quality of life in a cohort of healthy adult subjects. Eur Spine J 2016;25:3675–86. 27432430 10.1007/s00586-016-4702-2

[23] De Rezende Pratali R De Oliveira Luz C Barsotti CEG. Analysis of sagittal balance and spinopelvic parameters in a Brazilian population sample. Coluna/Columna 2014;13:108–11.

[24] Lee CS Chung SS Kang KC. Normal patterns of sagittal alignment of the spine in young adults radiological analysis in a Korean population. Spine (Phila Pa 1976) 2011;36:1648–54. 10.1097/BRS.0b013e318216b0fd 21394071

[25] Mac-Thiong JM Roussouly P Berthonnaud E. Age- and sex-related variations in sagittal sacropelvic morphology and balance in asymptomatic adults. Eur Spine J 2011;20: (Suppl 5): 572–7. 10.1007/s00586-011-1923-2 PMC3175918 21833574

[26] Kumar M Baklanov A Chopin D. Correlation between sagittal plane changes and adjacent segment degeneration following lumbar spine fusion. Eur Spine J 2001;10:314–9. 11563617 10.1007/s005860000239 PMC3611507

[27] Umehara S Zindrick MR Patwardhan AG. The biomechanical effect of postoperative hypolordosis in instrumented lumbar fusion on instrumented and adjacent spinal segments. Spine (Phila Pa 1976) 2000;25:1617–24. 10870136 10.1097/00007632-200007010-00004

[28] Mehta VA Amin A Omeis I. Implications of spinopelvic alignment for the spine surgeon. Neurosurgery 2015;76:707–21. 25692368 10.1227/01.neu.0000462077.50830.1a

[29] Franklin D Cardini A Flavel A. Morphometric analysis of pelvic sexual dimorphism in a contemporary Western Australian population. Int J Legal Med 2014;128:861–72. 24789357 10.1007/s00414-014-0999-8

[30] Zech WD Hatch G Siegenthaler L. Sex determination from os sacrum by postmortem CT. Forensic Sci Int 2012;221:39–43. 22521792 10.1016/j.forsciint.2012.03.022

